# Development and Application of 3D Bioprinted Scaffolds Supporting Induced Pluripotent Stem Cells

**DOI:** 10.1155/2021/4910816

**Published:** 2021-09-13

**Authors:** Dezhi Lu, Yang Liu, Wentao Li, Hongshi Ma, Tao Li, Xiaojun Ma, Yuanqing Mao, Qianqian Liang, Zhenjiang Ma, Jinwu Wang

**Affiliations:** ^1^Shanghai Key Laboratory of Orthopaedic Implants, Department of Orthopaedic Surgery, Shanghai Ninth People's Hospital, Shanghai Jiao Tong University School of Medicine, Shanghai 200011, China; ^2^School of Medicine, Shanghai University, Shanghai 200444, China; ^3^Spine Institute, Shanghai University of Traditional Chinese Medicine, Shanghai 200032, China; ^4^Department of Orthopedics, Shanghai General Hospital, Shanghai Jiao Tong University School of Medicine, Shanghai 200080, China

## Abstract

Three-dimensional (3D) bioprinting is a revolutionary technology that replicates 3D functional living tissue scaffolds in vitro by controlling the layer-by-layer deposition of biomaterials and enables highly precise positioning of cells. With the development of this technology, more advanced research on the mechanisms of tissue morphogenesis, clinical drug screening, and organ regeneration may be pursued. Because of their self-renewal characteristics and multidirectional differentiation potential, induced pluripotent stem cells (iPSCs) have outstanding advantages in stem cell research and applications. In this review, we discuss the advantages of different bioinks containing human iPSCs that are fabricated by using 3D bioprinting. In particular, we focus on the ability of these bioinks to support iPSCs and promote their proliferation and differentiation. In addition, we summarize the applications of 3D bioprinting with iPSC-containing bioinks and put forward new views on the current research status.

## 1. Introduction

The lack of tools for assessing promising drug targets impedes the development of treatments for various conditions, such as spinal cord injury and cardiovascular diseases. In addition, in some critical circumstances, such as organ transplantation, it is difficult to overcome the limited supply of suitable organ donors and a possible immune response to the organ transplant [1]. Therefore, approaches that enable tissue engineering for the development of innovative and effective treatments have been receiving increasing attention.

Three-dimensional (3D) bioprinting systems are promising tools for generating functional organs or tissues, which can be used for studying tissue morphogenesis, therapeutic drug screening, and possible organ regeneration in the future [2]. Bioprinting permits highly precise and accurate fabrication of biological 3D constructs containing cells, extracellular matrix scaffolds, and biochemical factors. Such 3D constructs can better mimic the human *in vivo* microenvironment than 2D cell culture environments and animal models [3].

Nevertheless, it has been reported that some cells may be damaged during the printing protocol due to shear stress [4]. Therefore, suitable bioinks and bioprinters need to be chosen to support the cells. Popular bioinks include some naturally derived biomaterials, such as polysaccharides, fibrin, and collagen, which have excellent biocompatibility. In addition to the natural scaffolds, some synthetic components can be also designed with tunable mechanical and degradation properties.

Various adult cells, such as mesenchymal stem cells, chondrocytes, cardiomyocytes (CMs), and endothelial cells, can be used for bioprinting. However, there are many limitations in the application of these adult cells in the repair of tissues and organs or the construction of *in vitro* models. First, bioprinting requires a large number of cells, and adult cells have a limited ability to proliferate. Secondly, autologous cells are difficult to obtain, whereas allogeneic cells may be rejected by the immune system.

Human-induced pluripotent stem cells (hiPSCs) have attracted global attention since their development a decade ago [5]. Because of their self-renewal properties and potential for multilineage differentiation, iPSCs may be used to generate a large number of autologous adult cells. Furthermore, the use of iPSCs allows to avoid ethical problems associated, for example, with the use of embryonic stem cells (ESCs) in stem cell research and various biomedical applications. In addition, using iPSCs to reconstruct tissues and organs in vitro better simulates normal and pathological processes, and thus, these cells serve as better research models. A noticeable challenge pertaining to the use of iPSCs in bioprinting is the multivariate nature of the differentiation process. Therefore, apart from minimizing the exposure to stress and harmful forces during the printing process, it is also important to provide the appropriate culture conditions to fabricate the desired differentiated cellular products [3]. There has been a significant interest in mimicking the 3D cytoarchitecture of native tissues *in vitro* and developing bioactive, biocompatible, and mechanically tunable 3D-configured bioinks that can be used for stem cell research and therapy [6].

Considering that several reviews devoted to 3D bioprinting of hiPSCs have been published recently, in the present review, we focus on different bioinks prepared by 3D bioprinting and discuss their advantages in supporting and promoting hiPSC proliferation and differentiation. In addition, we discuss the complex 3D printed microstructures developed to more closely mimic human physiology. We also review the use of hiPSCs in combination with 3D bioprinting for the treatment of neurological, orthopedic, cardiovascular, and hepatic disorders. Finally, in our concluding remarks, we summarize these recent advances and provide an outlook for the future development of these methods.

## 2. Popular Bioinks Supporting hiPSC Growth

HiPSCs can be seeded onto or cast within various supporting biomaterials. In addition, via 3D bioprinting, constructs containing hiPSCs can be directly fabricated by using a single-step approach to generate 3D cellularized scaffolds. Ho et al. describe neural induction of PU (a thermo-responsive synthetic hydrogel) encapsulated human hiPSCs, neither reported neurite outgrowth nor cell functionality [7, 8]. In the latter case, iPSCs can be closely integrated with biomaterials by encapsulation for direct and complete contact with the extracellular elements that mimic the native cell microenvironment. Some of the reported novel bioinks are presented below (Table 1).

However, compared with adult cells, such as chondrocytes, bone marrow mesenchymal stem cells, and endothelial cells, iPSCs, especially undifferentiated ones, are more sensitive. Depending on the bioprinting method used, the cells are exposed to high shear forces, radiation-induced damage, and electric or thermal stress during the printing process. All these factors have a marked impact on the proliferation and differentiation potential of iPSCs; thus, it is critical to choose the appropriate bioinks and bioprinting technology.

Koch et al. conducted several studies to investigate the effect of biomaterials on cell survival, pluripotency, and differentiation [12]. The laser bioprinting process itself had only a minor effect on cell survival. Survival rates of hiPSCs were higher if they were grown in Matrigel, blood plasma, and hyaluronic acid than in alginate, fibrin, collagen, or Geltrex. Notable cell apoptosis within 10 h may be ascribed to dissociation-induced cell death. After 24 h, high cell survival rates were observed when bioink medium or fibrinogen solution was used, each mixed with hyaluronic acid to achieve appropriate viscosity for bioprinting and avoid rinsing the cells. Importantly, the properties of hiPSCs within sols or gels were different from the characteristics of hiPSCs on the surface of bioinks.

Polysaccharide-based bioinks that contain polysaccharides, such as alginate, agarose, and chitosan, are widely used because they exhibit favorable biocompatibility, require mild cross-linking conditions, and have limited impact on iPSC properties. A polysaccharide-based bioink consisting of agarose, alginate, and carboxymethyl-chitosan was designed to support hiPSC expansion and differentiation in the clinical setting. Then, this bioink was cross-linked in calcium chloride to obtain a stable and porous construct. Such printed construction favored homogenous distribution and high viability of hiPSCs. After gelation, iPSCs could be maintained as self-renewing stem cells within the printed bioinks, and cell proliferation persisted for more than 9 days [14]. Shu et al. fabricated a 3D printed alginate tube structure (approximately 13 mm tall) composed of sodium alginate (1.5% *w*/*v*) and containing 600 mM (6%) of calcium chloride solution prepared in Millipore water. They showed that hiPSCs could be bioprinted with their valve-based printing process without adverse effects on their viability and pluripotency [15]. However, many of these polysaccharides are markedly fragile, lacking sufficient mechanical strength to be retained in the transplant tissue site, and often experiencing low mechanical properties.

### 2.1. Fibrin-Based Bioinks

Fibrin is a hydrogel formed by the enzymatic reaction between thrombin and fibrinogen—the key proteins involved in blood clotting—which supports extensive cell growth and proliferation [16]. Recently, fibrin-based bioinks have also been used as naturally derived biomaterials for supporting cultured stem cells and their differentiation into specific tissues [17, 18]. Sharma et al. designed a novel fibrin-based bioinks combined with drug-releasing guggulsterone microspheres and hiPSC-derived neural progenitor cells. The construct was printed as domes with a 1 cm diameter using a microfluidics-based RX1 bioprinter. Guggulsterone microspheres were used for controlled drug release; they also facilitated the survival of hiPSCs in bioprinted tissue and supported hiPSC differentiation into dopaminergic neurons [10]. The rapid degradation rate of pure fibrin is a major limitation for its use in differentiating hiPSCs for tissue engineering applications. Therefore, Robinson et al. designed a fibrin-based bioinks containing genipin, a natural cross-linking agent. They showed that genipin improved neuronal differentiation of neural progenitors derived from hiPSCs in 2D culture, having a concentration-dependent effect on the morphology and mechanical performance of 3D fibrin scaffolds [19]. The optimal concentration of genipin for its maximal neurotrophic and fibrin cross-linking effects ranged between 1 and 2.5 mM. Ruchi et al. show how our novel fibrin-based bioink formulation combined with drug releasing microspheres can serve as a tool for bioprinting tissues using hiPSC. They demonstrate that use a microsphere-laden bioink to bioprint hiPSC can promote the differentiation of neural tissue [20].

### 2.2. Hyaluronic Acid-Based Bioinks

Differentiation of stem cells into specific lineages during development is tightly regulated by the local microenvironment that includes growth factors, extracellular matrix proteins, and surrounding cells. Hyaluronic acid is one of the main components of the extracellular matrix, and hybrid scaffolds composed of hyaluronic acid and collagen can be used for cartilage regeneration. As the collagen concentration increases, the tensile strength performance of the scaffolds improves, and their degradation period increases [21]. Kupfer et al. optimized a photocrosslinkable formulation of native extracellular matrix proteins to promote the differentiation of iPSCs into CMs and used this bioink for the 3D printing of hiPSC-laden structures with two chambers and a vessel inlet and outlet [22].

### 2.3. Bioinks Based on Synthetic Components

In addition to the naturally derived biomaterials, synthetic components have been studied with respect to their utility as components of 3D printed structures. Li et al. investigated the feasibility of printing hiPSCs in a primed state after dissociation into single cells by using an extrusion-based 3D bioprinting method. The new bioink material, hydroxypropyl chitin, was introduced and mixed with bioactive Matrigel to form bioinks with different composition ratios [11]. Kerscher et al. designed a synthetic bioinks, consisting of polyethylene glycol and fibrinogen, which could be used to encapsulate hiPSCs, guide their differentiation, and promote the process of functional cardiac tissue formation [23]. Compared with bioinks composed of natural biomaterials or purely synthetic materials, such hybrid composite bioinks may have both good biocompatibility and tunable mechanical properties. Kerscher et al. designed two alginate/gellan gum/laminin (ALG/GG/LAM) hydrogel blends that are presented for the fabrication of hiPSC-based 3D neural models. Due to their wide range of applications, adjustability, and printing capabilities, the ALG/GG/LAM-based 3D neural models are of great potential for 3D neural modeling in the future [24].

## 3. Effect of the Micro/Nano Structure on hiPSC-Derived 3D Printed Tissue Model

Recently, novel methods have been used to fabricate complex 3D printed microstructures that could mimic human physiology more closely. Sophisticated 3D scaffold arrangements or micropatterning by 3D bioprinting favor tissue meddles to achieve high predictability and low cost efficiency [25].

**Figure 1:**
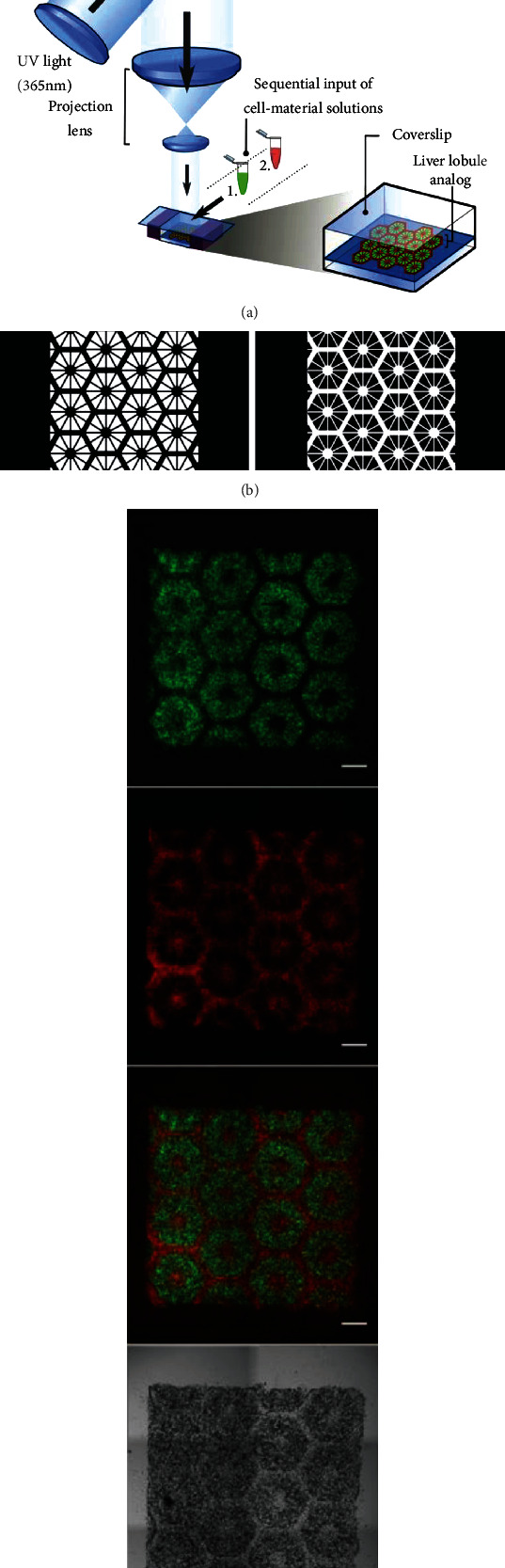
3D bioprinting of a hydrogel-based hepatic construct with complex 3D printed microstructure that more closely mimics the physiological properties of the human liver [9].

Ma et al. presented a patterned biomimetic hiPSC-derived hepatic model with microscale hexagonal units of liver cells and supporting cells created by using a 3D bioprinting method (Figure 1) [9]. First, 5% (wt/vol) gelatin methacrylate and 2% (wt/vol) glycidyl methacrylate-hyaluronic acid at a 1 : 1 ratio were chosen to support the cells. Then, the micropatterns, which mimicked the hepatic lobule structure, were transferred to hydrogels using the digital light processing-based 3D bioprinting method. To photopolymerize the hydrogel solutions, a digital micromirror device chip was used to generate photo-masks according to the input digital patterns. The entire process was completed in several seconds under minimal UV illumination. Compared with the features of the 2D monolayer culture, this 3D biomimetic liver model had improved morphological organization and exhibited increased secretion of the metabolic product, upregulated expression of the liver-specific genes, and enhanced cytochrome P450 induction in hiPSC-derived hepatic progenitor cells (HPCs).

**Figure 2:**
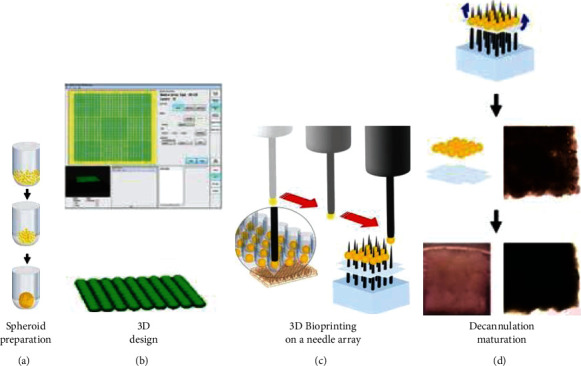
Schematic overview of biomaterial-free cardiac 3D bioprinting process [13].

**Figure 3:**
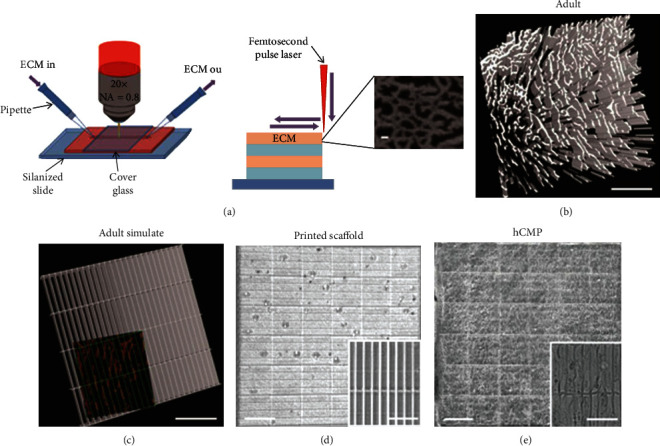
Human-induced pluripotent stem cell-derived cardiac muscle patch (hCMP) fabrication by 3D multiphoton excited printing [27]. ECM: extracellular matrix.

Ong et al. created biomaterial-free cardiac tissue using hiPSC-derived CMs by using a 3D bioprinting method [13] (Figure 2). In a sterile environment, the cardiospheres were picked up, transferred, and loaded onto a needle array individually by a robotic arm, in exact spatial coordinates with the help of specific 3D design software. Using this method, cardiospheres could be positioned in precise coordinates and flexible grid configurations, with uniform shape and thickness, which enabled to obtain a more uniform tissue structure at both the macroscopic and microscopic levels than afforded by random self-assembly of cardiospheres [26]. Park et al. also developed 3D cardiac tissue derived from hiPSCs by using 3D bioprinting on a needle array (Figure 3). Importantly, they built a computational model to visualize the 3D stress distribution to investigate the mechanical reliability of implantable tissues. Their method could serve as a nondisruptive framework that measures the contractility, beating patterns, and viscoelasticity of implantable cardiac tissues to be used in heart tissue regeneration [1].

To solve the problem of producing structures of the size at which individual cells interact, the multiphoton-excited 3D printing method was used to fabricate native-like extracellular matrix bioscaffolds with submicron resolution [27]. The scaffolds were seeded with smooth muscle cells, endothelial cells, and CMs (at a 1 : 1 : 2 ratio), which had been differentiated from hiPSCs to generate hiPSC-derived cardiac muscle patches.

## 4. 3D Bioprinting of iPSCs in Tissue Engineering

In recent years, multiple sources of iPSCs and biomaterials have been used in combination with bioprinting techniques in tissue engineering. The field of iPSC bioprinting is evolving rapidly.

### 4.1. Applications for Nerve Tissue Engineering

Millions of people worldwide are affected by nerve injuries that cause incapacitation due to permanent cognitive impairment, movement disabilities, and psychiatric problems, leading to increased economic burdens for the society and reduced quality of life [28, 29]. The emerging tissue-engineered nerve grafts can replace traditional nerve anastomosis as a treatment for nerve injury [30]. Several studies have attempted to understand the molecular pathogenesis of neurological disorders. hiPSC bioprinting technologies bring a change in the strategy of treating the structural damage to the nervous system. During the past decade, stem cells have been shown to have a potential for the repair of the impaired nervous system [31]. The iPSCs can be differentiated into neural crest or neural progenitor cells, which can subsequently be patterned to different neuron subtypes including glutamatergic, GABAergic, cholinergic, and dopaminergic neurons [32–34]. Stem cells secrete large amounts of cytokines and growth factors to induce the activity of local tissue progenitors and stromal cells, thereby promoting tissue repair [35].

**Figure 4:**
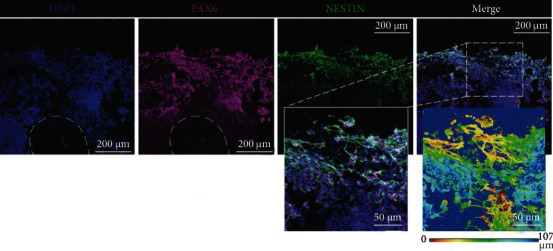
Immunophenotyping of 3D bioprinted hiPSCs 20 days after printing, including 17 days of neural induction [12].

3D printing of the iPSC-laden bioinks generated scaffolds containing consistently distributed stem cells. Bioinks including iPSCs, alginate, carboxymethyl chitosan, and agarose were squeezed and printed by Gu et al. [14] (Figure 4). The cells encapsulated in bioinks were continuously viable, the proportion of dead cells was almost negligible, and their activity remained high by prolonging in excess of 7 days. During the process of maintaining constructs for stem cell expansion, hiPSCs formed aggregates that generated large spheroids on day 7. This spherical state was similar to colony formation in the conventional 2D culture. Interestingly, the spheres in the 3D culture system contained tightly packed cell clusters, which were markedly different from the sharp, flat edges, and tight accumulation during the conventional 2D culture. In addition, immunophenotype identification by confocal microscopy showed that iPSC spheroids within the constructs expressed OCT4, SOX2, SSEA4, and TRA-1-60. Consequently, iPSCs *in situ* differentiated into embryoid bodies containing endoderm, ectoderm, and mesoderm cells. With the addition of neural induction/differentiation mediators, iPSC constructs can produce more homogeneous nerve tissue with neurons and supporting neuroglia. Minoru et al. develop an hiPSC-sensory neuron (SN) laden bioinks using highly purified and functional SN populations to 3D bioprint microarchitecture wirings that demonstrate responsiveness to warm/cold sense-inducing chemicals and mechanical stress. By a bioprinting technique, the hiPSC-SNs were seeded into the hollow microchannels created by sacrificial gelatin ink printed in the GelMA supporting bath; this biofabrication approach could be amenable to incorporate sensible SN networks into the engineered skin equivalents that have the potential to regenerate sensible functions by connecting host neuron systems in injured areas [36].

### 4.2. Applications for Bone Tissue Engineering

Osteoarthritis was once considered a degenerative disease of the joint. In recent years, it has been shown that this disease is caused by mechanical trauma, inflammation, and other factors that cause cartilage lesions. Cartilage components eliciting an autoimmune response cause secondary articular cartilage destruction [37, 38]. The mechanism is summarized as follows: (1) inhibition of proteoglycan synthesis and destruction of collagen fibers induce loss of articular cartilage elasticity, (2) increasing hydraulic permeability leads to increase compressive stress on chondrocytes, and (3) decomposing enzymes further destroy and reform the surface of articular cartilage [39, 40].

At present, autologous chondrocyte implantation and autologous cartilage tissue transplantation techniques are widely used in clinical practice. Although chondrocyte implantation can regenerate hyaline cartilage-like tissues, the mechanical function and durability of the newly generated articular surface are still suboptimal. Owing to the difference in thickness, texture, structure, and biomechanical properties of the cartilage, there are still problems with respect to selecting the chondrocyte donor area to adapt to the repair of cartilage defects in different parts of the joint [41, 42]. In addition, patients often need to perform surgical operations twice, and healing frequently depends on the quality and quantity of the autologous cells of the patient.

Tissue engineering of articular cartilage has made considerable progress. Bioprinted cartilage substitutes are used for the treatment of secondary knee osteoarthritis, articular joint injuries, and articular cartilage degeneration, which indicate their high clinical translation potential and demand [43, 44]. Mesenchymal stem cells can differentiate into cells of the mesodermal lineage, giving rise to numerous specialized connective tissues, including the bone, adipose tissue, and cartilage. In contrast to transplanted chondrocytes, mesenchymal stem cells preferentially differentiate into the bone. Concurrently, the composition of the bioinks in the process of biomanufacturing is not only important to ensure the long-term activity of hiPSCs and maintenance of the 3D structure but also to provide an appropriate physiological simulation environment after differentiation. Two bioinks have been recently investigated: nanofibrillated cellulose (NFC) with alginate (NFC/A) and NFC with hyaluronic acid (NFC/HA). The use of the NFC/A bioinks was associated with enhanced levels of cellular activity [45]. To induce directional cartilage differentiation, hiPSCs and irradiated mature chondrocytes were bioprinted in NFC/A and NFC/HA bioinks. After 5 weeks, hyaline-like cartilaginous tissue with collagen type II expression and lacking tumorigenic OCT4 expression was observed in 3D bioprinted NFC/A constructs. Moreover, a marked increase in cell number within the cartilaginous tissue was detected by 2-photon fluorescence microscopy, indicating the importance of high cell densities for achieving favorable survival after printing. It was concluded that NFC/A bioink was suitable for bioprinting iPSCs to support cartilage production in cocultures with irradiated chondrocytes. However, the time and concentration of chondrogenic factors need to be further optimized. In the future, the use of iPSCs for 3D bioprinting will be a potential treatment for repairing cartilage after joint damage. However, more studies are necessary to further optimize and improve the bioprinting scheme and obtain the functional proof of the newborn cartilage.

### 4.3. Application for Cardiovascular Tissue Engineering

Myocardial infarction seriously endangers human health. In the field of cardiovascular disease treatment, the fact that myocardial tissue lacks self-repairing ability is a serious challenge [46]. Traditional treatments, such as pharmacological therapy, angioplasty, and coronary artery bypass surgery, cannot restore necrotic and fibrotic damaged myocardium to the normal state [47, 48]. In recent years, it has been found that stem cell transplantation can repair diseased myocardium [49]. Since the very first engineered heart tissues were introduced more than two decades ago, a wide array of hiPSC-derived cardiac spheroids, organoids, and heart-on-a-chip models has been developed incorporating the latest available technologies and materials [50].

hiPSCs have emerged as a key component of cardiac tissue engineering. Scaffold-free cellular spheroids obtained from a coculture of hiPSC-derived CMs, fibroblasts, and endothelial cells were 3D printed, and these cardiac cellular patches were tested successfully in rat models of myocardial infarction [51]. Lui et al. think that hiPSC-derived CMs have been bioprinted to recapitulate a vascularized cardiac tissue which can then be transplanted in a defective heart [52]. Cho et al. used a 3D bioprinter to produce scaffold-free cardiac tissue grafts from hiPSC-derived CMs cell spheroids, and the results find that mechanical stretching stimulates hiPSC-derived CMs in a 3D printed, scaffold-free tissue graft to develop mature cardiac material structuring and cellular fates [53]. However, hiPSCs transplanted to lesions undergo oxidative damage and demonstrate squeeze loss as well as low cell survival and retention rates [54]. The development of biological 3D printing technology may overcome these problems to some extent. hiPSCs derived from the peripheral tissues of patients with disease specific mutations are a valuable tool to study the cardiac pathophysiology and drug development. The hiPSC-derived cardiac cells were successfully used to model cardiac diseases such as dilated cardiomyopathy and myocardial infarction [55]. The disease models help to identify the cellular phenotypes critical to cardiac pathology [56, 57].

Kupfer et al. used this bioink to 3D print hiPSC-laden structures with two chambers and a vessel inlet and outlet. After hiPSCs proliferated to a sufficient density, they differentiated the cells within the structure and demonstrated function of the resultant human chambered muscle pump. This advance represents a critical step toward generating macroscale tissues; human chambered organoids of this type might also serve as a testbed for cardiac medical devices and eventually lead to therapeutic tissue grafting [58].

When hiPSCs are separated into single cells, they are very sensitive to the process. Their pluripotency and directional differentiation ability are affected by many external factors, including printing methods, biological material, culture medium, and cell density [12]. A recent study used laser-assisted bioprinting technology to study the sensitivity of hiPSCs to biomaterials, such as collagen, alginate, hyaluronic acid, fibrinogen, fibrin, Geltrex ™, Matrigel ™, and cell culture medium. The results showed that bioinks with 85% E8 and 15% hyaluronic combined with Matrigel as a coating substrate provided optimal conditions for the survival and growth of hiPSCs [12]. Moreover, the study found that hiPSCs were not sensitive to laser printing itself, and cell survival was improved, and pluripotency was maintained after printing. In addition, directed differentiation showed that when hiPSCs were printed and induced to differentiate into CMs, they started to beat, demonstrating their functional cardiac phenotype. Some studies using hiPSCs derived from CMs to print directly [59]. Such studies described a method to produce photocrosslinkable tissue-specific decellularized extracellular matrix (dECM) bioinks for fabricating patient-specific tissues with high control over complex microarchitecture and mechanical properties achieved by a digital light processing-based scanningless and continuous 3D bioprinter. They demonstrated that tissue-matched dECM bioinks provided a conducive environment for maintaining high viability and maturation of hiPSC-derived CMs and hepatocytes.

**Figure 5:**
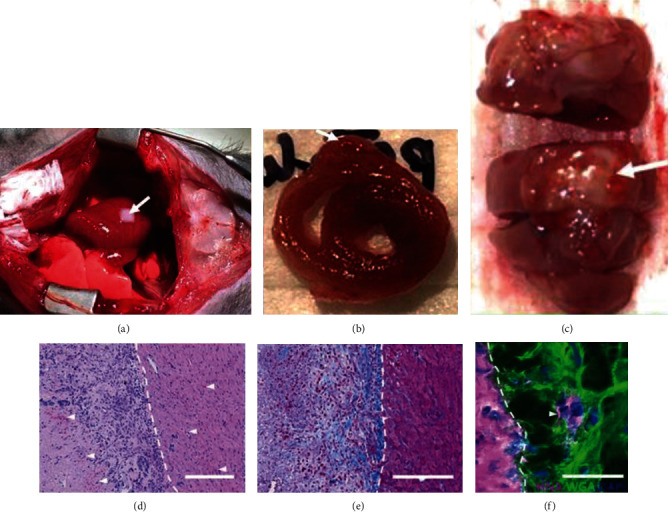
*In vivo* implantation of 3D bioprinted cardiac patches [13].

In addition, there were some interesting studies in the field of cardiovascular engineering that did not use bioinks. Recently, a protocol was established to obtain iPSC-derived endothelial progenitors (endothelial colony-forming cells) and smooth muscle-forming cells to assemble into vascular cell spheroids [60]. In these constructs, a layered distribution of alpha smooth muscle actin-positive cells and extracellular matrix deposition were achieved [13, 61]. The same biological manufacturing method can also generate heart tissue. One study used hiPSC-derived vascular cell spheres as a novel cell material for scaffold-free biological construction. The spheres were composed of mixed human umbilical vein endothelial cells and hiPSC-derived CMs. Using this cell sheet technology, cardiac tissue sheets were generated, and the heart showed signs of vascularization when cardiac tissue sheets were implanted in rat hearts [13] (Figure 5). More research should be conducted to further study the construction of complex cardiac structures and functions of cardiovascular tissue by screening suitable biomaterials and improving the printing methods.

### 4.4. Applications for Liver Tissue Engineering

Liver transplantation is the only effective treatment for the end-stage liver disease. At present, the 5-year survival rate after liver transplantation has reached 73.6%. However, the number of patients waiting for liver transplantation worldwide far exceeds the number of donors. The shortage of donor livers has become an important factor restricting the development of liver transplantation [62]. In-depth studies of liver regeneration and the developments in cell culture, stem cell technology, and genetic engineering should permit liver printing in the near future. One example is that researchers bioprinted hepatic tissue constructs using iPSC-derived hepatocytes, endothelial cells, and mesenchymal cells resuspended in two different bioinks: GelMA with stiffness similar to healthy liver tissues and a mix of glycidyl methacrylate-hyaluronic acid/GelMA which supported vascularization [63, 64]. However, liver printing has two crucial bottlenecks: the need to recreate a complicated internal system of branched blood vessels and the difficulty of distributing more than three high-density functional cells in a 3D structure and making them grow into tissues.

Chapin and Hajjar first attempted to produce iPSC-derived liver bioprinting models. In their study, HLCs differentiated from both hiPSCs and hESCs were bioprinted and examined for the presence of hepatic markers to further validate the compatibility of the valve-based bioprinting process with the transfer of fragile cells [15]. The results showed that the examined cells were positive for nuclear factor 4 alpha, secreted albumin, and had morphology that was similar to that of hepatocytes. Both hESC and hiPSC lines were tested postprinting, and it was found that printed and nonprinted cells had negligible differences in terms of viability and pluripotency. To solve the bottlenecks of complex microstructures and different cell combinations in the liver microenvironment, one study used 3D bioprinting technology to construct a human liver model with a microstructure composed of hexagonal liver cell units and supporting cells that reflected structural and physiological features of the liver lobular array [9]. hiPSC-HPCs were embedded with human umbilical vein endothelial cells and adipose-derived stem cells in a microscale hexagonal architecture. This improved the functional properties and conformation of hiPSC-HPCs. Liver marker expression analysis showed that HPC maturity in the construct increased. To develop more physiological bioinks, this team mixed photocrosslinkable gelatin methacrylate and pig liver dECM. By adjusting the mechanical properties of the liver dECM bioink formula, the cell phenotype in the bioprinting construct could be improved, and the complex biochemical components of the liver phenotype were suitably maintained, which promoted the differentiation and maturation of hepatocytes derived from hiPSCs [59]. Liver organ printing technology has considerable potential and can provide patients with a specific platform for pathophysiology research, early drug screening, and clinical transformation.

### 4.5. Other Applications

In other study, a porous grid-type hiPSC-derived mesenchymal stem cell- (hi-MSC-) loaded hydrogel scaffold was constructed using a 3D bioprinting device. The 3D printed hydrogel scaffold provided a permissive in vitro living environment for hi-MSCs and significantly increased the survival duration of transplanted hi-MSCs when compared with hi-MSCs administered locally in vivo. This study confirms that 3D printed hi-MSC-loaded scaffold not only promoted the recovery of the endometrial histomorphology (endometrial tissue and gland regeneration) and the regeneration of endometrial cells (stromal cells and epithelial cells) and endothelial cells but also improved endometrial receptivity functional indicators [65]. In addition, hiPSCs that were generated from anterior cruciate ligament were used in the repair of ligaments and tendons [66].

## 5. Conclusions

Biological preparation of 3D printed stem cells is still a relatively novel research field, and various protocols for their manufacturing are in their infancy (Table 2). The clinical application of 3D printed stem cells has a long way to go and faces many challenges. The main reasons for the limited clinical application of these cells to date are related to the 3D printing methods, selection of biological materials, and acquisition of stem cells. The basic problems include printing speed, accuracy, survival rate, and cell viability. Bioinks as carriers should not only have optimal biocompatibility, degradability, and mechanical properties but also be suitable for mass production. The optimization of printing methods is another key aspect in achieving translation into the clinic.

The use of 3D printed iPSCs in the medical field is still in its preliminary stage. It has marked potential for use in tissue engineering and repair. Current research is mainly concentrated in the fields of orthopedic, neural, hepatic, and cardiovascular regeneration. Based on the continuous optimization of printing methods and biological inks, the use of multidirectional differentiation of the cells can achieve precise repair of complex structures. Simultaneously, the potential harm caused by multidirectional differentiation is also a point to be considered in future studies.

In summary, with the rapid development of stem cell 3D printing technology, biomaterials suitable for stem cell 3D printing are critical to its clinical application. Identification and validation of such materials will provide strong support for the realization of organ printing in the future and help solve the shortage of organ donors.

## Figures and Tables

**Table 1:** Novel 3D printed bioinks and their applications in tissue engineering.

Bioinks	Applications	Bioprinting methods	References
Gelatin methacrylate (5% wt/vol) + glycidyl methacrylate-hyaluronic acid + hiPSCs	Liver tissue	Digital light processing-based 3D printing	Ma et al. [9]
Fibrin (20 mg/mL), alginate (5 mg/mL), and genipin (0.3 mg/mL) + hiPSCs	Neural tissue	Microfluidics-based RX1 bioprinter	Abelseth et al. [10]
Hydroxypropyl chitin + bioactive Matrigel + hiPSCs	3D microtissue differentiation and drug screening	Extrusion-based 3D bioprinting	Li et al. [11]
Agarose + alginate + carboxymethyl chitosan + iPSCs	Neural tissue	3D bioprinting	Gu et al. [12]
hiPSC-derived CMs + human umbilical vein endothelial cells + human adult ventricular cardiac fibroblasts to form mixed cell spheroids	Cardiac patches	3D bioprinter	Ong et al. [13]

**Table 2:** 3D bioprinting of iPSCs in tissue engineering applications.

Application fields	Cell sources	Bioprinting outcomes	References
Nerve tissue engineering	iPSCs	Embryoid bodies containing endoderm, ectoderm, and mesoderm cells	Gu et al. [12]
Bone tissue engineering	iPSCs	Cartilage	Nguyen et al. [45]
Cardiovascular tissue engineering	iPSCs	CMs	Koch et al. [23]
	iPSCs	CMs/hepatocytes	Yu et al. [59]
	hiPSCs, HUVECs, HCFs	Cardiac tissue sheets	Ong et al. [13]
	iPSCs	Vascular cells	Moldovan et al. [60]
Liver tissue engineering	iPSCs, hESCs	Hepatic markers	Jones et al. [14]
	iPSCs	Hepatic markers	Ma et al. [9]
	iPSCs	Hepatic markers	Yu et al. [59]

iPSCs: induced pluripotent stem cells; HCFs: human cardiac fibroblasts; hESCs: human embryonic stem cells; HUVECs: human umbilical vein endothelial cells.
